# Effects of Visual Stimulation with Bonsai Trees on Adult Male Patients with Spinal Cord Injury

**DOI:** 10.3390/ijerph14091017

**Published:** 2017-09-05

**Authors:** Hiroko Ochiai, Chorong Song, Harumi Ikei, Michiko Imai, Yoshifumi Miyazaki

**Affiliations:** 1 Department of Plastic and Reconstructive Surgery, National Hospital Organization Tokyo Medical Center, Higashigaoka 2-5-1, Meguro-ku, Tokyo 152-8902, Japan; ochiroko@gmail.com; 2 Center for Environment, Health and Field Sciences, Chiba University, 6-2-1 Kashiwa-no-ha, Kashiwa, Chiba 277-0882, Japan; crsong1028@chiba-u.jp (C.S.); ikei0224@ffpri.affrc.go.jp (H.I.); 3 Forestry and Forest Products Research Institute, 1 Matsunosato, Tsukuba, Ibaraki 305-8687, Japan; 4 Le Verseau Inc., 3-19-4 Miyasaka, Setagaya-ku, Tokyo 156-0051, Japan; leverseau@mvb.biglobe.ne.jp

**Keywords:** spinal cord injury, nature therapy, bonsai trees, visual stimulation, near-infrared spectroscopy, heart rate variability

## Abstract

Nature therapy has been demonstrated to induce physiological relaxation. The psychophysiological effects of nature therapy (stimulation with bonsai trees) on adult male patients with spinal cord injury (SCI) were examined. Oxyhemoglobin concentration changes in the prefrontal cortex were measured using near-infrared spectroscopy, and heart rate variability was analyzed. Psychological responses were evaluated using the modified semantic differential method and Profile of Mood States (POMS) subscale scores. Visual stimulation of adult male patients with SCI elicited significantly decreased left prefrontal cortex activity, increased parasympathetic nervous activity, decreased sympathetic nervous activity, increased positive feelings, and resulted in lower negative POMS subscale scores. Nature therapy can lead to a state of physiological and psychological relaxation in patients with SCI.

## 1. Introduction

Stress appears to be increasingly present in our modern and demanding industrialized society. Every aspect of our bodies and brains can be virtually influenced by stress induced by living in an urban environment [1]. Early human civilizations lived in natural settings, demonstrating that we can adapt to nature. Congruent with this viewpoint, individuals living in modern societies who are experiencing stress have become interested in several types of natural therapy [2]. For example, psychological evaluations of the effects of horticultural therapy on the elderly have been previously reported [3,4].

The progress and development of research involving nature and forest medicine has advanced in recent years with the development of medical equipment related to the natural and life sciences. For example, oxyhemoglobin (oxy-Hb) concentrations in the prefrontal cortex were measured using a portable near-infrared spectroscopy (NIRS) device, which revealed that foliage plants can have physiological relaxation effects in male participants [5]. Heart rate variability (HRV) was measured, which revealed that visual stimulation with roses increased parasympathetic nervous activity [6] and that fresh pansies decreased sympathetic nervous activity [7]. Furthermore, salivary cortisol levels were measured after the participants were subjected to gardening activity, which demonstrated decreased stress levels [8]. A review by Song et al. presented scientific data to elucidate the physiological relaxation effects of nature therapy on the activities of the central nervous system, autonomic nervous system, endocrine system, and immune system [9]. The results from these experiments are based on advances in various physiological indicators from the viewpoint of evidence-based medicine in Japan. Nature therapy has the potential to be more widely adopted as preventive medicine in the future.

One potential application of nature therapy is its use for patients with spinal cord injury (SCI). SCI is a devastating event for individuals, and they frequently develop motor and sensory impairments, as well as autonomic dysfunction. Previous studies have reported that autonomic nervous activity plays a major role in social cognition and that difficulties in the ability to interpret social information are commonly observed in a variety of mental disorders, which in turn correlate with poor autonomic nervous system regulation [10]. Depressive disorders are the most frequent concern following SCI and significantly affect rehabilitation, community integration, quality of life (QOL), and health-related outcomes [11,12,13]. A clinical practice guideline published in 1998 noted that 25% of men and 47% of women with SCI experienced some form of depressive disorder [14].

Considering this high prevalence of psychological distress, it is especially important to highlight that according to research, most patients with SCI felt that their emotional needs were not sufficiently addressed by their rehabilitation team [15]. Recent meta-analyses have reported medium-to-large effect sizes for psychological interventions for post-SCI depression, and there is sufficient evidence specifically supporting the use of cognitive behavioral therapy interventions [16]. However, these methods require the intervention of an expert psychiatrist.

An advantage of nature therapy such as viewing bonsai trees is that it allows for routine, self-induced mental relaxation. Such therapy is also accessible for individuals who are unable to perform certain activities (e.g., walking more than a mile or doing vigorous activities). If the physical and mental stress of patients with SCI can be reduced via intervention with nature therapy, this therapy can be recommended to such patients to promote improved health. Similarly, nature therapy can be used as a preventive medicine therapy for healthy but stressed individuals. Relaxing effects have been reported regarding exposure to forest, urban green space, flowers and plants, and so on. Nature therapy is defined as “a set of practices aimed at achieving ‘preventive medical effects’ through exposure to natural stimuli that render a state of physiological relaxation and boost the weakened immune functions to prevent disease” [9]. 

Although previous analytical studies have pointed out the relevance of nature therapy and relaxation in healthy adults, there is no previous research on adult patients with SCI. To the best of our knowledge, this is the first study to examine the physiological and psychological effects of nature therapy in adult male patients with SCI and clarify its effectiveness in reducing daily stress.

In this study, 24 Japanese adult male patients with chronic-stage SCI were exposed to 10-year-old cypress bonsai trees as visual stimuli. Bonsai is miniature natural landscapes in pots using trees and other plants. They are a famous art form unique to Japan.

## 2. Materials and Methods

### 2.1. Experimental Design

All participants gave their informed consent for inclusion before they participated in the study. The study was conducted in accordance with the Declaration of Helsinki, and the protocol was approved by the Ethics Committee of the Center for Environment, Health and Field Sciences, Chiba University, Japan (Project identification code number: 5). In total, 24 Japanese male patients with spinal cord injury aged 25–79 years (mean age, 49.0 ± 16.4 years) were included in this study. They had a height of 162–182 cm (171.4 ± 5.6 cm) and weight of 52–94 kg (67.2 ± 9.2 kg). The patients had no psychiatric disorders, which comprised part of the inclusion criteria for the study, and they were in the chronic stage of their condition (i.e., >1 year after the lesion developed). They were diagnosed with spinal cord injury by a doctor and their damage was located below C7. The patients were able to independently move around in wheelchairs.

The experiments were conducted in an experimental room at Chiba University. The room temperature was maintained at 23.7 ± 1.3 °C, and the relative humidity was maintained at 50.5 ± 7.4%. The patients moved into the experimental room and the experiments were separately carried out for each patient.

**Figure 1:**
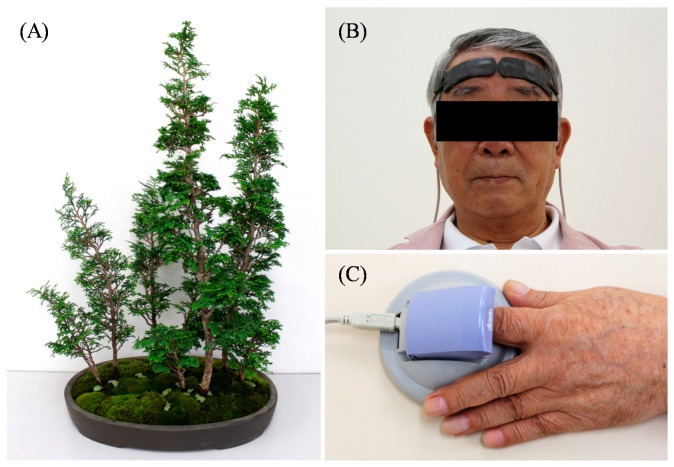
Bonsai trees and physiological measurement apparatuses. (**A**) Japanese cypress bonsai trees; (**B**) Participant undergoing near-infrared spectroscopy (NIRS) measurement; (**C**) Participant undergoing heart rate variability (HRV) measurement.

Miniature potted 10-year-old Japanese cypress bonsai trees were used as visual stimuli. Eight cypress trees, approximately 55 cm in height, were grouped together in a 40 × 20 × 5 cm ceramic pot (Figure 1A). Before visual stimulation, these miniature trees were covered by a corrugated cardboard box (rest condition). After a 60-s rest period, the patients viewed the miniature potted trees (visual stimulation) or nothing (control) for 60 s each; all patients were made to experience both experimental conditions. Distance from the patients’ eyes to the trees was 60–63 cm. The order of conditions (i.e., visual stimulation vs. control) was randomized. The patients practiced the procedure, using visual stimulation with a potted plant, once beforehand.

### 2.2. Physiological Indices

Changes in oxy-Hb concentrations on the surface of the prefrontal cortex were measured using a two-channel near-infrared spectroscopy device (NIRS; Pocket NIRS Duo, DynaSense, Hamamatsu, Japan). NIRS probes were placed bilaterally and symmetrically on the forehead. Two sensors were placed over the frontal region, with one sensor placed on the left side of the forehead and the other placed on the right side of the forehead (Figure 1B) [17]. To analyze the NIRS response, change in the oxy-Hb concentrations in the prefrontal cortex during visual stimulation was measured. The difference between this and the value from 10 s prior to stimulation was analyzed. It is well established that oxy-Hb concentration reflects the activation of neural regions [18].

The patients placed their left forefingers on the sensor of an accelerated plethysmograph (ARTETT, U-Medica Inc., Osaka, Japan) (Figure 1C). Heart rate variability (HRV) was analyzed. HRV was converted by a 60/a-a interval; the sampling frequency was 1000 Hz. The power levels of the high-frequency (HF) (0.15–0.40 Hz) and low-frequency (LF) (0.04–0.15 Hz) components were calculated using the maximum entropy method [19,20]. HF power was considered to reflect parasympathetic nervous activity, and the LF to HF ratio was considered to reflect sympathetic nervous activity [21,22]. In general, parasympathetic nervous activity is enhanced during relaxation and sympathetic nervous activity is enhanced at the time of awakening or in situations of stress.

### 2.3. Psychological Indices

The modified semantic differential (SD) method and Profile of Mood States (POMS) subscale scores were used to evaluate psychological responses following visual stimulation. The modified SD method uses three pairs of adjectives anchoring 13-point scales: “comfortable to uncomfortable,” “relaxed to awakening,” and “natural to artificial” [23]. The scores were determined for the following six POMS subscales: “tension-anxiety (T-A),” “depression (D),” “anger-hostility (A-H),” “vigor (V),” “fatigue (F),” and “confusion (C).” A short form of POMS with 30 questions was used to decrease participant burden [24,25,26]. The “total mood disturbance (TMD)” score was calculated by [(T-A + D + A-H + F + C) –V]. A high TMD score indicates an unfavorable psychological state.

### 2.4. Statistical Analysis

We used paired *t*-tests to compare physiological indices and the Wilcoxon signed-rank test to compare psychological test scores. All statistical analyses were performed using SPSS version 20.0 (IBM Corp., Armonk, NY, USA). Data are expressed as means ± standard error (mean ± SE). For all cases, *p* < 0.05 (one-sided) was considered statistically significant. One-sided tests were used because we hypothesized that the patients would be relaxed after viewing the bonsai trees.

## 3. Results

**Figure 2:**
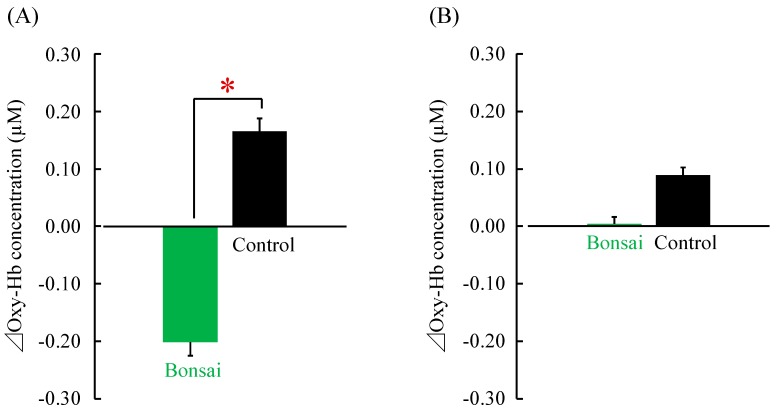
Mean oxy-Hb concentrations in the prefrontal cortices. (**A**) Changes in the left prefrontal cortex when viewing bonsai trees vs. control; (**B**) Changes in the right prefrontal cortex. *N* =  24, mean ± standard error. * *p* < 0.05, paired *t*-test. Δ, change.

Oxy-Hb concentrations of the left and right prefrontal cortices were measured using a two-channel NIRS device. Change in the oxy-Hb concentration of the left prefrontal cortex was significantly lower when the patients viewed the bonsai trees (visual stimulation) than when they viewed nothing (control) (visual stimulation = −0.20 ± 0.02 μM; control = 0.17 ± 0.02 μM; *p* < 0.05; Figure 2A). Oxy-Hb concentration of the right prefrontal cortex did not significantly differ between visual stimulation (0.00 ± 0.01 μM) and control (0.09 ± 0.01 μM; Figure 2B) conditions.

**Figure 3:**
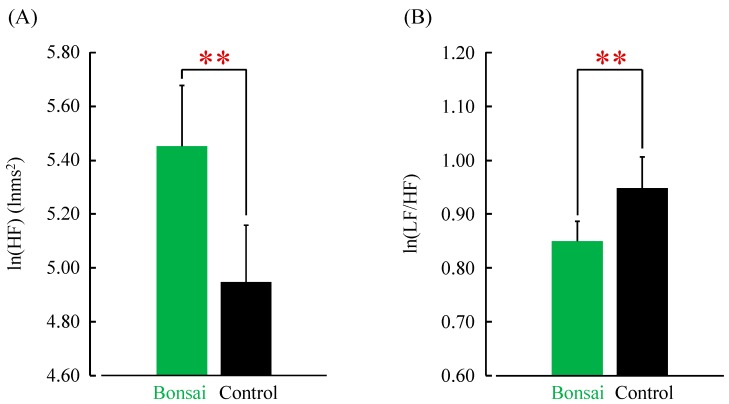
Autonomic nervous activity when viewing bonsai trees vs. control. (**A**) Parasympathetic nervous activity: mean natural logarithm (ln) of the high-frequency (HF) component; (**B**) Sympathetic nervous activity: mean natural logarithm (ln) of the ratio of low-frequency (LF) to HF (LF/HF). *N* = 24, mean ± standard error. ** *p* < 0.01, paired *t*-test.

The average power of the high-frequency (HF) components of HRV, which is related to parasympathetic nervous activity, increases when we feel relaxed [21,22]. This value was significantly greater when the patients viewed the bonsai trees compared with the control condition (visual stimulation = 5.45 ± 0.23 lnms^2^; control = 4.95 ± 0.21 lnms^2^; *p* < 0.01; Figure 3A). The average low-frequency (LF) to HF ratio of HRV, which is related to sympathetic nervous activity, increases when we feel stressed [21,22]. This ratio was significantly lower when the patients viewed the bonsai trees compared with the control condition (visual stimulation = 0.85 ± 0.04; control = 0.95 ± 0.06; *p* < 0.01; Figure 3B). 

**Figure 4:**
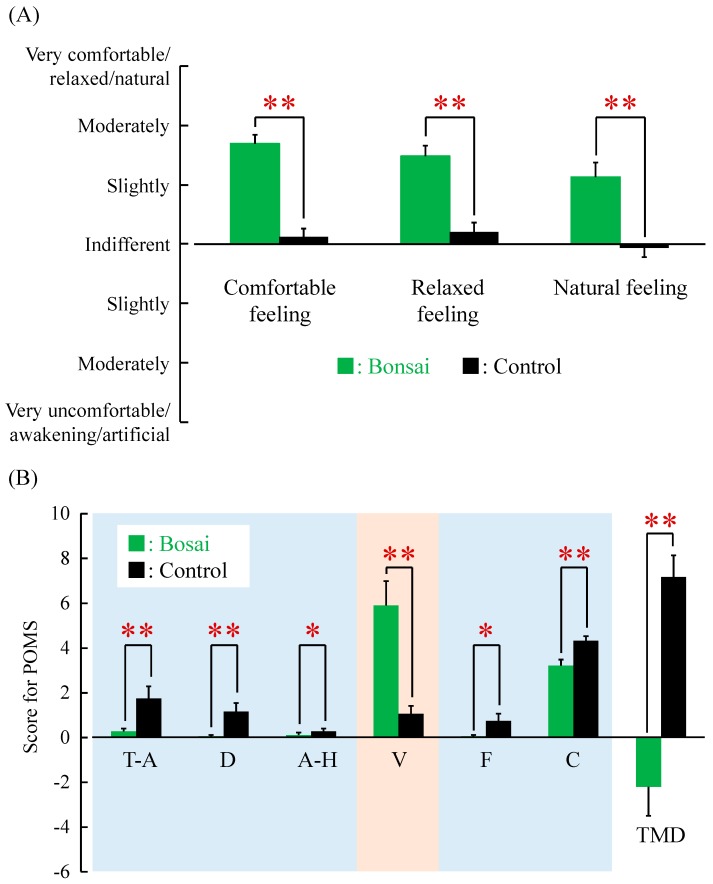
Questionnaire results. (**A**) Subjective feelings measured using the modified semantic differential method after viewing the bonsai trees vs. control. *N* = 24, mean ± standard error. ** *p* < 0.01, Wilcoxon signed-rank test; (**B**) Scores on the Profile of Mood States after viewing the bonsai trees vs. control. T-A, tension-anxiety; D, depression; A-H, anger-hostility; V, vigor; F, fatigue; C, confusion; and TMD, total mood disturbance. *N* = 19, mean ± standard error. * *p* < 0.05 and ** *p* < 0.01, Wilcoxon signed-rank test.

Figure 4A shows the results of the modified SD (semantic differential) method. Subjective evaluations indicated that the patients felt significantly more “comfortable,” “relaxed,” and “natural” when viewing the bonsai trees compared with the control condition (*p* < 0.01). The Profile of Mood States (POMS) was used to gauge the patient’s psychological response to stimuli (Figure 4B). Negative POMS subscale scores of “tension-anxiety,” “depression,” “confusion,” “anger-hostility,” and “fatigue” were significantly lower when viewing the bonsai trees compared with the control condition (*p* < 0.05). On the other hand, the scores of “vigor,” a positive subscale, were significantly higher when viewing the bonsai trees compared with the control condition (*p* < 0.01). The scores of global “total mood disturbance” were significantly lower when viewing the bonsai trees compared with the control condition (*p* < 0.01); indeed, negative emotions were significantly reduced when the patients were exposed to natural stimuli.

## 4. Discussion

Research demonstrates that oxygen consumption, regional cerebral blood response, and oxy-Hb supply are increased in highly activated neural regions [18]. A lower oxy-Hb concentration indicates that the quantity of oxygen transmitted to the prefrontal cortex tissue is small. The lower prefrontal cortex activity found in the current study is consistent with that reported in previous studies [27,28], showing that low oxy-Hb concentrations represent the calming of brain activity. For example, in the dorsolateral prefrontal cortex, hemispheric specialization of emotional processing has been proposed by functional magnetic resonance imagining. In particular, the activation of the left prefrontal cortex has been associated with positive mood and the processing of positive stimuli, whereas the activation of the right prefrontal cortex has been linked to negative mood and the processing of negative stimuli [29]. However, the precise details of the function of the right and left prefrontal cortex as measured using the NIRS device remain unknown. In this experiment, the activity of the left prefrontal cortex was suppressed, whereas that of the right prefrontal cortex did not change when the patients viewed the bonsai trees. Based on these results, we can only conclude that the patients were in a relaxed state when they viewed the bonsai trees.

Patients with SCI have reduced autonomic flexibility, as measured using HRV, and exhibit reduced autonomic modulation during emotion recognition tasks [10]. However, in the current study, the patients with SCI showed significantly higher parasympathetic nervous activity and significantly lower sympathetic nervous activity when exposed to a natural stimulus. These results demonstrate that in patients with SCI, the autonomic nervous system responds to natural stimuli in a similar manner to that in healthy adults [9,30].

Patients with SCI report pain-related disability, depression, fatigue, pressure sores, spasticity, and issues with bladder and bowel management [31]. These conditions often induce negative mood states in patients with SCI; thus, emotional support is an important factor influencing the rehabilitation process [32]. The focus of rehabilitation for such patients has shifted from medical management to QOL issues, and exposure to natural stimuli represents one way to improve QOL in patients with SCI.

Viewing bonsai trees simulates “forest therapy,” a therapeutic activity that has become popular in Japan, and utilizes the scientifically proven effects of walking through and viewing forests [33,34]. Indeed, forest therapy is increasingly recognized as a relaxation and stress management strategy with demonstrated clinical efficacy. Forest therapy suppresses sympathetic nervous activity, increases parasympathetic nervous activity, and reduces cortisol levels and cerebral blood flow in the prefrontal cortex [9]. Forest therapy has also been shown to increase human natural killer cell activity and improve immunity [35,36,37], and these effects have been proven to last for at least seven days [36,37]. In addition, psychological studies have demonstrated the benefits of forest environments on subjective measures of stress, cognitive function, and mood [38]. There is a difference between forest therapy and this experiment; namely, one is performed in the field and the other indoors. The field experiment reveals the general influence of nature on humans through their five senses. On the other hand, we can pick up the effect of stimulating a single sense in the indoor experiment and clarify its influence. Here, we chose vision as one of the senses that we use in the forest, and we clarified the influence of visual stimulation by bonsai trees.

In the current study, the same psychophysiological effects of visual stimulation with bonsai trees were shown in adult male patients with SCI as in healthy adults. We consider it an important point that a relaxation effect could also be obtained in SCI patients by applying nature therapy, because it is difficult to perform forest therapy with SCI patients with restricted activities. Further, these findings may help promote the development of the environment, which is beneficial to the physical and mental health of individuals with disability.

The main limitation of the present study was its small sample size. Also, the results of the present study cannot yet be extrapolated to females. On the other hand, we compared only the condition of viewing miniature potted trees (visual stimulation) with viewing nothing (control) in this study, but it would be useful to investigate a third condition with a non-nature object. Furthermore, a short-term stimulus was used; the results were compared before and after only 60 s of stimulation with bonsai trees. In addition, we do not know for how long the effect of this natural therapy may last. Future studies examining the duration of effects following exposure to natural stimuli are required.

## 5. Conclusions

In conclusion, the current study revealed that visual stimulation with bonsai trees in adult male patients with SCI elicited the following: (1) significantly suppressed left prefrontal cortex activity; (2) significantly increased parasympathetic nervous activity and decreased sympathetic nervous activity; (3) significantly increased “comfortable,” “relaxed,” and “natural” feelings as assessed using the modified SD method; and (4) significantly decreased negative and increased positive POMS subscale scores. The findings of this study can be applied to SCI patients by taking advantage of their natural surroundings to ensure improved health and reduced mental stress. Generally, considerably less practice is supported by research, and the reality is that little research is applied in practice. We can say that this study is useful because in practice the effects are proved by research data.
